# A Case of Traumatism

**Published:** 1892-03

**Authors:** George A. Hermance

**Affiliations:** Avon, N. Y.


### A CASE OF TRAUMATISM.

George A. Hermance, M. D., Avon, N. Y.

March 4th, 1889.—Elsie C., aet. six. A blue-eyed, light-haired child of very active nature. On February 22d, while running across the floor, fell and struck her forehead with terrific force, upon the floor. An hour or two after, she complained of feeling cold, mostly in her back, which external warmth failed in any way to relieve. After a restless night, nausea and vomiting appeared in the morning, also a chill at about ten o'clock, which was followed by fever. At noon the temperature was $103\frac{3}{4}$, and she complained of great pain in her forehead. Later in the day, extreme sensitiveness of the back of the neck and spine appeared, accompanied by photophobia and intolerance of all noise. There was an aggravation of the fever between 9 and 12 A. M., and 5 and 8 P. M., during which time her face would be flushed, and the cheeks a very dark red. As the case developed, there was an ineffectual urgency to stool, and dark, scanty urine, which later became paler and more profuse. When she was moved she begged the nurse not to let her head drop.

On the fourth day there was no improvement, and I saw the patient was showing symptoms of failure. She then presented the following symptoms: Temperature, $103\frac{1}{2}$; cheeks, dark red and burning to touch; starts from sleep with confused cries, it taking some moments to pacify her; screams from the slightest touch or movement of the bed or her body; begs not to have her head dropped when it is necessary to move her body; lies with body rigid, with head slightly thrown back; great photophobia; voice has a sharp and unnatural sound; she is imperious in her demands, wants very cold water almost constantly, probably would drink much at a time were it allowed her.

At this point I expressed my desire for consultation, and Dr. Nisbet, a very fair-minded and unprejudiced member of the old school was called in to give a diagnosis, after which competent homœopathic advice was to be secured. It was at 10.30 A. M. that I saw the patient as above described, and I gave Hypericum$^{6x}$ in water every two hours. At 5 P. M. Dr. Nisbet saw the case with me, and imagine my surprise, if not discomfiture, at our finding the temperature down to 101½, no sensitiveness of the spine, no photophobia, and the patient allowing herself to be handled without a word of objection. My discomfiture was due to the fear that I had made the case out to be worse than it was, and would meet with ridicule. When Dr. Nisbet pronounced it a case of *malaria*, I said nothing, but endeavored to get my scattered wits together sufficiently to make out just what was the matter, and what had taken place anyway. Here was a child that a few hours earlier was in a condition sufficiently serious to alarm me, now kicking her leg about, and joyfully telling her mother that the light did not hurt her eyes any more. The improvement continued, and she was on placebos next day. Dr. Nisbet said he would give Quinine, but knowing that I was a "Biegler homœopath" that I would stick to the teachings of Hahnemann, he said: "Give whatever you have for malaria, and you will cure your case." I had given Arnica, Bell., and Nux-v. before the Hypericum. My diagnosis was traumatic cerebro-spinal meningitis.

